# SHARED AND DISTINCT ASSOCIATIONS OF MANUAL DEXTERITY AND GROSS MOTOR FUNCTION WITH BRAIN ATROPHY

**DOI:** 10.1093/geroni/igad104.1170

**Published:** 2023-12-21

**Authors:** Ryan Dougherty, Hang Wang, Alden Gross, Jennifer Schrack, Yuri Agrawal, Eleanor Simonsick, Susan Resnick, Qu Tian

**Affiliations:** Johns Hopkins University, Baltimore, Maryland, United States; Johns Hopkins University, Baltimore, Maryland, United States; Johns Hopkins University Bloomberg School of Public Health, Baltimore, Maryland, United States; Johns Hopkins University, Baltimore, Maryland, United States; Johns Hopkins School of Medicine, Baltimore, Maryland, United States; National Institute on Aging, National Institutes of Health, Baltimore, Maryland, United States; National Institute on Aging, National Institutes of Health, Baltimore, Maryland, United States; National Institute on Aging, National Institutes of Health, Baltimore, Maryland, United States

## Abstract

Poor motor function is associated with brain volume and cognitive impairment. Less is known about the associations among motor domains and brain atrophy and whether associations are affected by cerebrovascular burden and/or physical activity. We analyzed data from 726 Baltimore Longitudinal Study of Aging participants (mean age 70.6±10.1 years, 56%women, 27%Black), 525 of whom had repeat MRI scans over 5.0±2.1 years. Two motor domains were operationalized as latent variables. Manual dexterity using the Purdue Pegboard and gross motor function using grip strength and comprehensive physical performance testing. Associations between the latent variables and cortical and subcortical brain volumes of interest were examined using latent growth curve modeling, adjusted for demographics, white matter hyperintensities, and physical activity. Both higher manual dexterity and gross motor function were cross-sectionally associated with smaller ventricular volume and greater white matter volumes in the frontal, parietal, and temporal lobes (all p<.05). Higher manual dexterity was also cross-sectionally associated with greater parietal gray matter ( =0.14;95%CI:0.05,0.23), hippocampal ( =0.10;95%CI:0.01,0.20), postcentral gyrus ( =0.11;95%CI:0.01,0.20), and occipital white matter ( =0.10;95%CI:0.01,0.21) volumes, and better gross motor function with greater temporal gray matter volume ( =0.16;95%CI:0.05,0.26). Longitudinally, higher manual dexterity and gross motor function were associated with lower temporal white matter and occipital gray matter atrophy (all p<0.05). Greater manual dexterity was also associated with a slower rate of ventricular enlargement ( =-0.17;95%CI:-0.29,-0.05) and less atrophy of occipital white matter ( =0.39;95%CI:0.04,0.71). Among cognitively normal middle- and older-aged adults, manual dexterity and gross motor function exhibited shared as well as distinct associations with brain atrophy over time.

